# NAC transcription factors ATAF1 and ANAC055 affect the heat stress response in Arabidopsis

**DOI:** 10.1038/s41598-022-14429-x

**Published:** 2022-07-04

**Authors:** Nouf Owdah Alshareef, Sophie L. Otterbach, Annapurna Devi Allu, Yong H. Woo, Tobias de Werk, Iman Kamranfar, Bernd Mueller-Roeber, Mark Tester, Salma Balazadeh, Sandra M. Schmöckel

**Affiliations:** 1 Department of Biochemistry, Faculty of Science, King Abdulaziz University, Jeddah, Saudi Arabia; 2 Division of Biological and Environmental Sciences and Engineering (BESE), King Abdullah University of Science and Technology (KAUST), Thuwal, Saudi Arabia; 3 Department Physiology of Yield Stability, Institute of Crop Science, University of Hohenheim, Fruwirthstr. 21, 70599 Stuttgart, Germany; 4 Max Planck Institute of Molecular Plant Physiology, 14476 Potsdam-Golm, Germany; 5 Institute of Biochemistry and Biology, University of Potsdam, 14476 Potsdam‐Golm, Germany; 6 Center of Plant Systems Biology and Biotechnology (CPSBB), 139 Ruski Blvd., 4000 Plovdiv, Bulgaria; 7 Institute of Biology, Leiden University, Sylviusweg 72, 2333 BE Leiden, The Netherlands; 8 Present Address: Department of Biology, Indian Institute of Science Education and Research (IISER), Tirupati, India

**Keywords:** Plant sciences, Plant stress responses, Heat

## Abstract

Pre-exposing (priming) plants to mild, non-lethal elevated temperature improves their tolerance to a later higher-temperature stress (triggering stimulus), which is of great ecological importance. ‘Thermomemory’ is maintaining this tolerance for an extended period of time. NAM/ATAF1/2/CUC2 (NAC) proteins are plant-specific transcription factors (TFs) that modulate responses to abiotic stresses, including heat stress (HS). Here, we investigated the potential role of NACs for thermomemory. We determined the expression of 104 Arabidopsis NAC genes after priming and triggering heat stimuli, and found *ATAF1* expression is strongly induced right after priming and declines below control levels thereafter during thermorecovery. Knockout mutants of *ATAF1* show better thermomemory than wild type, revealing a negative regulatory role. Differential expression analyses of RNA-seq data from *ATAF1* overexpressor, *ataf1* mutant and wild-type plants after heat priming revealed five genes that might be priming-associated direct targets of ATAF1: *AT2G31260* (*ATG9*), *AT2G41640* (*GT61*), *AT3G44990* (*XTH31*), *AT4G27720* and *AT3G23540*. Based on co-expression analyses applied to the aforementioned RNA-seq profiles, we identified *ANAC055* to be transcriptionally co-regulated with *ATAF1*. Like *ataf1*, *anac055* mutants show improved thermomemory, revealing a potential co-control of both NAC TFs over thermomemory. Our data reveals a core importance of two NAC transcription factors, ATAF1 and ANAC055, for thermomemory.

## Introduction

Temperatures higher than the plants’ adaptive thresholds cause heat stress (HS), which diminishes their growth, survival and productivity. Plants, by nature, have the ability to tolerate a certain degree of high temperature above their ambient temperature. This is known as ‘basal thermotolerance’. In addition to basal thermotolerance, plants also have the ability to acquire thermotolerance if they are pre-exposed to mild non-lethal higher temperatures, in a process called ‘heat priming’^1–4^. This heat priming induces not only an immediate response, but also leads to molecular and metabolic changes that persist for some time in the absence of the stress and that allow plants to respond more effectively to a second HS event. The second stress is known as the ‘triggering stimulus’^3,4^. The time period between priming and triggering stimuli is called the ‘memory phase’, during which stress memory can form and consolidate. Thermomemory refers to the maintenance of some, but not all, HS-induced changes, which ‘primes’, or prepares plants to respond more rapidly and strongly if such stress recurs before the memory fades. Experimental evidence indicates that thermomemory can range from several hours to days^4–6^ or even generations^7^. Thermomemory appears to be regulated, at least in part, by a set of genes different from those acting in basal thermotolerance and acquired thermotolerance^1,4,5,8–11^.

HS priming involves the activation of heat shock transcription factors (HSFs) that induce the expression of heat shock proteins (HSPs) which help to protect cellular proteins from denaturation and contribute to the repair or removal of misfolded proteins^12,13^. Several HSFs have been shown to be involved in the HS response^9,14–16^. In particular, class HSFA1s are considered as ‘master regulators’ of the HS response^16^, regulating the expression of several other transcription factor (TF) genes, including *DEHYDRATION RESPONSIVE ELEMENT BINDING PROTEIN 2A* (*DREB2A*), *HSFA2*, *HSFA7a*, *HSFBs*, and *MULTIPROTEIN BRIDGING FACTOR 1C* (*MBF1C*)^17^. While immediate responses to HS are relatively well studied, the molecular and physiological processes underlying thermomemory in plants are still not well understood. One of the possible mechanisms involves the accumulation of TFs in their inactive state after a priming stimulus (during the memory phase), and their activation upon experiencing the triggering stimulus^18,19^. For example, heat shock factor A2 (HSFA2) has been identified as a key component in the establishment of thermomemory in primed plants^9^. HSFA2 induces the expression of *HEAT STRESS-ASSOCIATED 32* (*HSA32*), which was found to be required specifically for the maintenance of thermomemory and to participate in the maintenance of cellular homeostasis during high temperature stress^8^.

NAM/ATAF1/2/CUC2 (NAC) is a family of plant-specific TFs that have an important role in the response to different biotic and abiotic stresses, including water deficit, salinity stress and temperature stress^20–22^. Several NACs have been reported to be involved in the basal HS response in *Arabidopsis thaliana* and crop plants^23–27^. For example, overexpression of *ANAC019* improved the thermotolerance of Arabidopsis, presumably by regulating the expression of *HSFA1s* and other *HSFs* including *HSFBs*^25^. The Arabidopsis membrane-associated NAC transcription factor gene *NTL4* has also been found to be responsive to heat stress, and *ntl4* mutants exhibited a higher cell viability and less H_2_O_2_ accumulation than WT, under HS^27^. In rice (*Oryza sativa*), expression of *SNAC3* is induced by several stresses, including heat, and its overexpression enhances tolerance to high temperature, drought and oxidative stress^26^. The expression of the *Triticum aestivum* (wheat) NAC transcription factor *TaNAC2L* is also induced by high temperature, and its overexpression in Arabidopsis improves its acquired heat tolerance via regulating expression of its heat stress-related genes^23^. However, there is limited evidence on the involvement of NAC TFs in regulating thermomemory. Arabidopsis JUNGBRUNNEN1 (JUB1) is the only NAC TF that has so far been reported to regulate thermomemory. *JUB1* expression is induced by HS and its expression pattern during the memory phase is similar to the expression pattern of other thermomemory associated genes, such as *HSFA2*^24^. Overexpression of *JUB1* improves the thermomemory of Arabidopsis seedlings^24^.

In this study, we set out to systematically identify NAC TFs responsive to thermomemory in Arabidopsis. We analyzed the expression patterns of nearly all (104) Arabidopsis NACs after priming HS, during the thermomemory phase, and after the triggering stimulus. One NAC identified in our screen is *ARABIDOPSIS TRANSCRIPTION ACTIVATOR FACTOR 1* (*ATAF1*; also called *ANAC002*). ATAF1 was previously identified to be involved in the gene regulatory networks of senescence, drought and sugar signaling^28–31^. We found that overexpression of *ATAF1* in transgenic Arabidopsis plants limits thermomemory capacity, while knocking out *ATAF1* profoundly enhances thermomemory and survival of plants exposed to a more severe HS. To identify genes regulated by ATAF1 in thermomemory, we employed RNA-seq to compare the heat responses in wild-type plants and mutants with altered *ATAF1* levels (overexpressors and knockout mutants). We found that *ATAF1* is co-expressed with *ANAC055*. With respect to thermomemory, the *ataf1*/*anac055* double-mutant displayed a phenotype similar to that of the single knock-out mutants *ataf1* and *anac055*, suggesting that the two TFs cooperatively regulate thermomemory.

## Results

### Identification of priming-responsive and memory-associated NAC transcription factors

**Figure 1:**
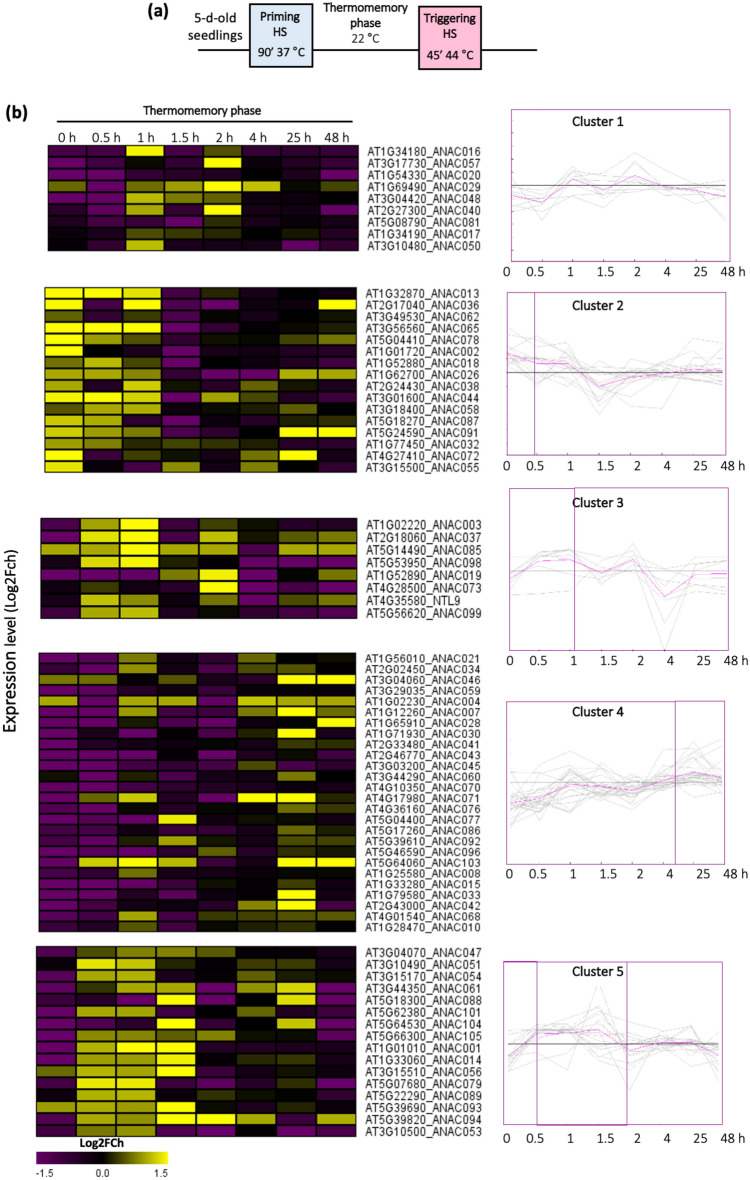
Expression of NAC transcription factors during the memory phase, after heat priming. (**a**) Schematic representation of the heat stress (HS) regime applied to asses HS memory. (**b**) Heat map and k-means clustering (k = 5 clusters) of NACs, based on their change in expression (HS samples compared to unstressed controls) during the memory phase at the time points indicated in the representation in (**a**). The color indicates the expression ratio as log2-fold change for the different time points, where ‘yellow’ denotes an increased expression and ‘purple’ a reduced expression. The list of genes in each cluster and their expression values are shown in Supplementary Table S1.

To investigate the expression pattern of NAC TFs in response to HS priming and during the memory phase, we treated Arabidopsis seedlings with a mild HS (priming) before exposing them to a severe HS (triggering stimulus). We collected samples at multiple time points after priming (up to 48 h into the thermomemory phase) and tested the expression of 104 NAC genes; seedlings kept in unprimed conditions were used as controls (Fig. 1).

Considering a 1.5-fold change as cut-off, 75 of the NACs were differentially expressed upon priming compared to control, while expression of 29 NAC genes was undetectable in the whole-seedling samples. The cut-off threshold was chosen considering that even moderate changes in TF expression levels can elicit strong downstream responses^32^. The expression of some NACs (including e.g., *ANAC013* and *ANAC029*/*AtNAP*) in response to HS priming was similar to that reported in a previous study by Kilian et al.^30^. NACs were grouped in clusters based on their expression pattern using k-means clustering. Changes in the expression of many NACs was already detectable immediately after the priming treatment (Fig. 1, Supplementary Table S1). Three clusters comprising 33 genes in total (clusters 1, 2 and 3) appeared upregulated during early timepoints, and largely unchanged during later timepoints (Fig. 1), while 26 genes (in cluster 4) were initially strongly downregulated, and then strongly upregulated during later time points (Fig. 1). For the genes in cluster 5 we observed a strong downregulation at time point 0, which is immediately after completion of the 90 min HS, followed by a strong upregulation wthin the time frame of up to 2 h after the HS, and then a moderate to unchanged expression (Fig. 1). Taken together, the NAC TFs analyzed exhibited different expression profiles during HS priming and memory, suggesting different roles in the response to HS.

### The effect of heat priming and triggering on the expression of NAC transcription factors

**Figure 2:**
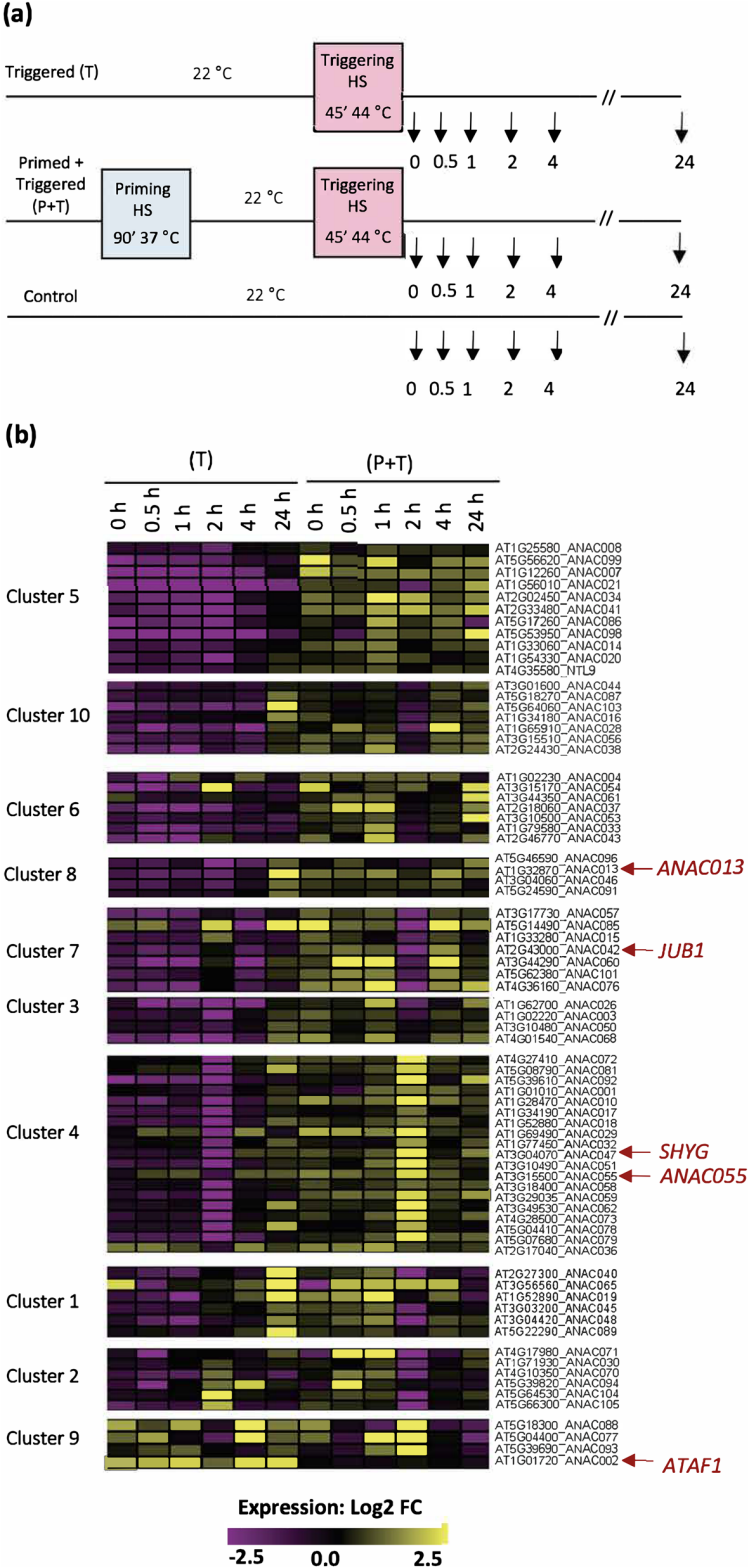
Expression of NACs after a triggering stimulus. (**a**) Schematic representation of the time points used for expression analysis of NACs in triggered plants (T), and in plants that were primed before triggering (P + T). In triggered (T) plants, the expression was calculated as a ratio of HS samples compared to unstressed controls. In primed and triggered (P + T) plants, the expression was calculated as primed and triggered (P + T), compared to triggered only (T). (**b**) K-means clustering of NAC expression profiles of primed and triggered (P + T) and triggered (T) plants after the triggering stimulus (k = 10 clusters). The expression of genes in each cluster is displayed as a heat map. The color indicates the expression as log2-fold change for the different time points, where yellow denotes an increased expression and purple a reduced expression. The list of genes in each cluster and their expression values are shown in Supplementary Table S2.

Pre-exposure of plants to a moderate stress (priming) alters their response to upcoming severe temperature stress. We tested this, at the molecular level, by investigating the transcriptional response of NAC TFs. We tested whether or not heat-priming affects the expression of NACs after the triggering HS. To this end, we determined the expression of NAC TFs after the triggering stimulus (T plants) and compared it with the expression in primed and triggered plants (P + T), using a 1.5-fold change cut-off. To identify specific expression patterns between P + T and T plants, a k-means clustering approach was applied, using 10 clusters (Fig. 2, Supplementary Table S2). We noticed that expression of most NAC TFs was induced in primed + triggered plants, compared with triggered-only plants. Only few genes (cluster 9) showed an opposite expression pattern, being mostly repressed in primed + triggered plants and induced in triggered-only plants (Fig. 2). *ATAF1* displayed a particularly interesting pattern: its expression remained almost unchanged in plants after priming + triggering (P + T), and expression was considerably induced in triggered-only conditions (without prior priming) (Fig. 2, cluster 9).

To test the hypothesis that ATAF1 is functionally involved in thermotolerance, additional physiological experiments were performed on *ataf1* mutants and plants overexpressing *ATAF1*. Two other genes from clusters 4 (*ANAC047*/*SHYG*, *AT3G04070*) and 8 (*ANAC013*, *AT1G32870*) were included for comparison.

### Functional analysis of selected NACs for thermomemory

**Figure 3:**
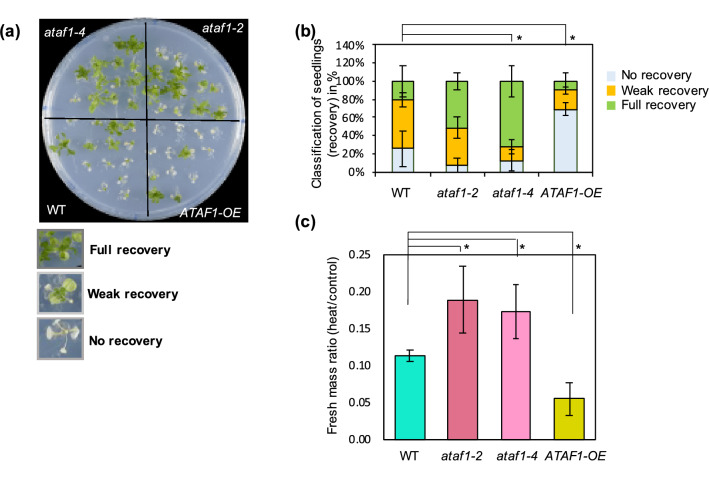
Improved thermomemory in *ataf1* mutants*.* (**a**) Thermomemory phenotype of *ATAF1* transgenic plants. Seedlings of *ATAF1-*OE, *ataf1-2*, *ataf1–4*, and WT were exposed to the HS regime schematically shown in Fig. 1a. Photos were taken 14 days after the second HS (triggering stimulus). The phenotype of one representative replicate of at least three independent biological replicates is shown. (**b,c**) Quantification of the results shown in (**a**). (**b**) Percentage of seedlings in different phenotypic classes; the thermotolerance phenotype was classified into three classes depending of the extent of plant recovery. The phenotype classes are illustrated in panel (**a**). (**c**) Seedling fresh mass in HS-primed and triggered plants compared to unstressed control plants. Error bars represent the standard deviation, calculated from three biological replicates; each replicate is the average mass of 13 seedlings. Significant differences between transgenic and WT plants were calculated using Student’s *t*-test; *indicates *P*-value < 0.05.

To investigate the impact of the selected NAC genes on thermomemory, we assessed their functional involvement in the process by analyzing the phenotype of overexpressor and knockout (or knockdown) mutants in response to a triggering HS given 3 days after heat priming (3 days of thermomemory). As shown in Supplementary Fig. S1, *ANAC013* and *SHYG* overexpressors, *shyg* knockout and *anac013* knockdown lines were not significantly different from wild type when compared in the thermomemory assay. However, *ATAF1* appeared to be involved in thermomemory. A phenotypic analysis of *ATAF1* transgenic plants for thermomemory showed a strong phenotype, where the *ataf1–2* and *ataf1–4* mutants had a significantly higher survival rate and fresh mass compared with WT plants, while plants overexpressing *ATAF1* (called *ATAF1*-OE in the following) exhibited a severely reduced thermomemory (Fig. 3a–c). When the *ataf1–4* mutant was transformed with an *ATAF1* allele (*pATAF1::ATAF1-GFP*) which expresses an *ATAF1-GFP* fusion from the native *ATAF1* promoter, thermomemory was restored to wild-type response (Supplementary Fig. S2). Taken together, these results suggest a negative regulatory role of *ATAF1* in thermomemory.

### Potential target genes of ATAF1

**Figure 4:**
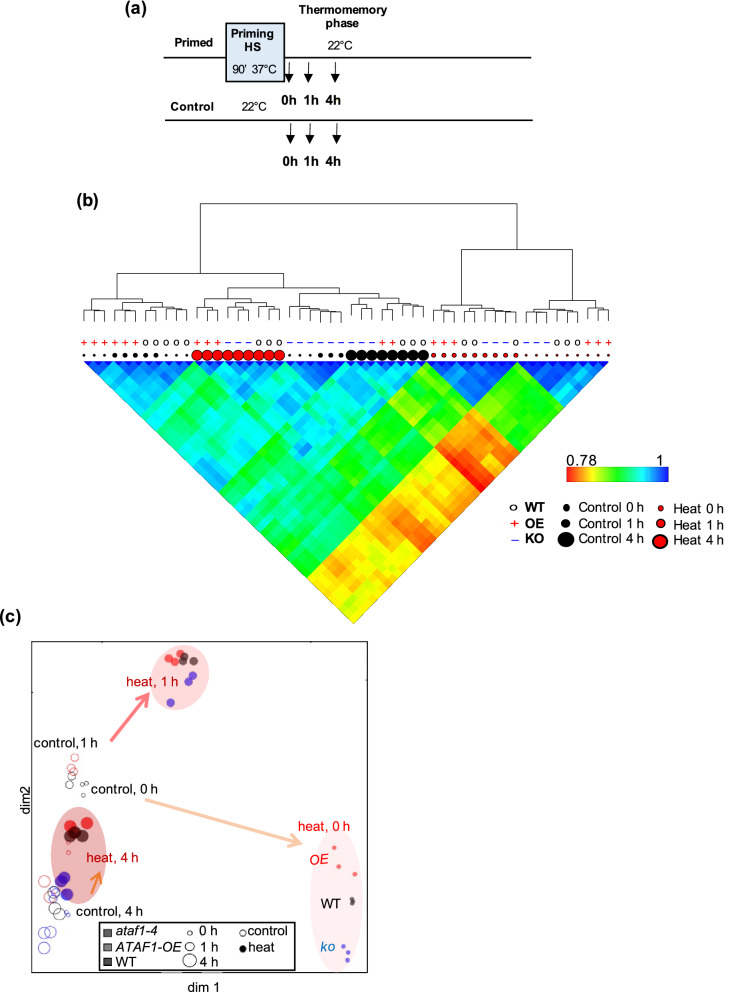
Clustering of RNA-seq data. (**a**) Schematic presentation of the time points used for expression analysis by RNA-seq. (**b**) Top: Hierarchical clustering of the samples according to their expression patterns. “+” denotes *ATAF1*-OE lines; “0” denotes wild-type Col-0; and “−” denotes *ataf1–4*. The sizes of the circles indicate the time elapsed since HS (0, 1, and 4 h after heat stress). The black and red colors indicate whether the plants underwent control or heat temperature treatment for 90 min. Legend: colored heat map of Pearson’s coefficients of expression profiles between all possible pairs of the samples. A partial rainbow color scheme, from red to blue, indicates the range of correlation values. (**c**) Multidimensional scaling (MDS) plot. Overall similarities and differences between samples, based on a multidimensional scaling analysis. Briefly, the Euclidean distance between all samples was calculated according to the expression levels of all genes, and represented in a two-dimensional space.

As our data suggested that *ATAF1* is a negative regulator of thermomemory we sought to understand the role of *ATAF1* in response to a heat priming stimulus by identifying its regulated target genes. To this end, WT, *ATAF1-*OE and *ataf1–4* mutant seedlings were treated with the priming stimulus (37 °C for 90 min), and samples were then collected at three time points (0 h, 1 h and 4 h; Fig. 4a) after the heat priming for gene expression profiling by RNA-seq. To eliminate the potential influence from other environmental factors, control samples (unprimed control) were collected at the same time points as the heat-treated samples.

To understand the relationship between samples in the RNA-seq, a hierarchical clustering analysis was performed and a heat map of the Pearson’s correlation coefficients of the expression profiles between all possible pairs of the samples was established (Fig. 4b). We observed a clustering between biological replicates, suggesting a high reproducibility of the experiment. The heat map also showed a strong separation between samples based on HS conditions, and by genotype (Fig. 4b). The overall similarities and differences between samples were confirmed by multidimensional scaling (MDS) (Fig. 4c), supporting a stronger separation by condition (heat and control) than by genotype. We also noticed a similarity of samples under control temperature and those after 4 h of heat stress, suggesting a rapid recovery of most changes in gene expression. Of note, the expression of *ATAF1* in WT seedlings during the memory phase, as revealed by RNA-seq, followed the pattern determined by qRT-PCR (Supplementary Fig. S3).

**Figure 5:**
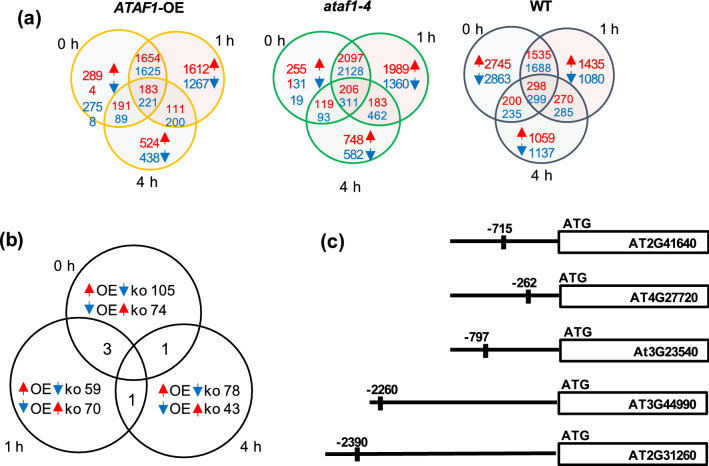
Global transcriptomic changes of thermomemory-associated genes potentially regulated by ATAF1. (**a**) Venn diagrams of heat-induced and heat-repressed genes, after heat priming (heat relative to control) in WT, *ataf1–4* mutant, and *ATAF1*-OE transgenic plants. (**b**) Venn diagram of genes upregulated in *ATAF1*-OE and downregulated in *ataf1* mutant, and vice versa, compared to WT, in response to a priming HS, after 0 h, 1 h or 4 h. (**c**) Schematic representation of the position of ATAF1-binding sites in the upstream regions of genes identified in (**b**).

For all three genotypes, we found the highest number of differentially expressed genes (DEGs) at time point 0, right at the end of the 90-min heat priming treatment (Fig. 5a). To identify priming HS-associated genes, we used the same criteria that were previously used by Sedaghatmehr et al.^4^. We investigated genes whose expression was induced after priming and remained high at all examined time points in the memory phase, as well as genes whose expression was down-regulated and remained low during the memory phase (Fig. 5a). Among the DEGs are thermomemory-associated *HSPs*^4^ whose expression in response to heat priming was similarly induced in *ATAF1*-OE, *ataf1–4* mutant and WT, such as *HSP22, HSP21, HSP17.4* and *HSP18.2* (Supplementary Table S3). This finding corroborates the conclusion that these HSPs contribute to a general heat-response, which is common to all three genotypes.

To identify differential responses of *ATAF1*-OE and *ataf1–4* mutant plants to heat priming, we analysed the RNA-seq data for genes upregulated in *ATAF1*-OE, compared to WT, but downregulated in *ataf1–4* mutant plants, and vice versa (Fig. 5b). Next, to identify potential direct target genes of ATAF1, we analyzed the promoters of differentially expressed genes for either the presence of the ATAF1 binding site (reported by Garapati et al.^31^) or for binding by ATAF1 as determined by DNA affinity purification sequencing (DAP-seq) experiments^33^. The analysis was performed for each time point after the heat priming (0 h, 1 h and 4 h). In total, we identified 64 genes as likely direct ATAF1 targets (Supplementary Table S4). We then refined our analysis by searching for those potential target genes that are commonly regulated by ATAF1 in all three, or two, time points after heat priming. Five genes, i.e., *AT2G31260* (*ATG9*), *AT2G41640* (*GT61*), *AT3G44990* (*XTH31*), *AT4G27720* and *AT3G23540* were commonly regulated at two of the three timepoints, suggesting they might be priming-associated direct targets of ATAF1 (Fig. 5c); no gene was regulated at all three time points. *ATG9* is an autophagy gene^34^ and experimental evidence indicates that autophagy plays a role in the heat stress response^35,36^. Its involvement in thermomemory is yet to be confirmed. The two genes *GT61* and *XTH31* are related to cell wall biosynthesis and expansion. Glycosyltransferase 61 (GT61) belongs to the glycosyltransferase (GT) family. GT proteins have diverse functions in plants, but most of them are likely involved in the biosynthesis of polysaccharides and glycoproteins in the cell wall. In grasses, including rice (*Oryza sativa*) and wheat (*Triticum aestivum*), GT61 family enzymes are involved in the synthesis of xylans, one of the main components of the cell wall^37–39^. GT61 has not yet been shown to be involved in heat stress priming or tolerance. XTH31 belongs to the xyloglucan endotransglucosylase/hydrolase (XTH) family. Generally, members of the XTH family are involved in cell wall remodeling, expansion and morphogenesis suggesting a potential role in stress responses^40^. In Arabidopsis, XTH31 is involved in regulating cell wall xyloglucan content^41^. The *xth31* loss-of-function mutant has a reduced sensitivity to ABA, and seeds germinate faster than those of the WT^41,42^. Transgenic soybean (*Glycine max*) plants overexpressing *XTH31* from Arabidopsis display enhanced tolerance to flooding along with more adventitious roots and longer primary roots^43^. The two genes *AT4G27720* and *AT3G23540* are not well characterized. *AT4G27720* is annotated to encode a major facilitator superfamily protein with a molybdate ion transporter function, while *AT3G23540* is annotated to encode a protein of the alpha/beta-hydrolase superfamily. The α/β-hydrolase enzymes are involved in various processes, including biosynthesis, metabolism, signal transduction, gene regulation^44^, and in the plants’ response and tolerance to salinity stress^45^.

### Co-regulatory network of ATAF1 reveals potential co-regulation with ANAC055

**Figure 6:**
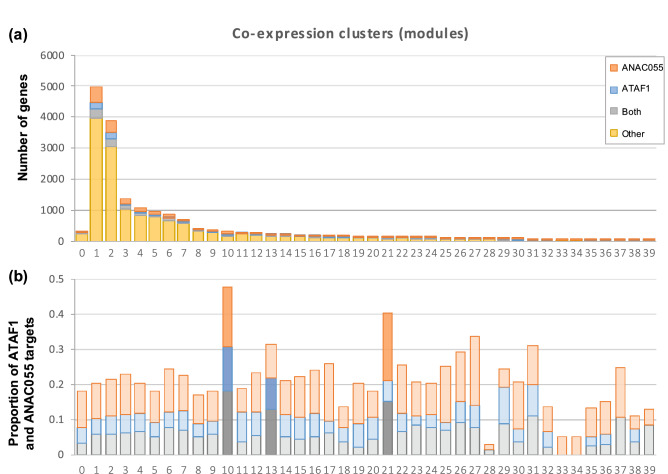
Potential targets of ATAF1 in response to heat stress. (**a**) The bar graph shows the number of genes in each co-expression cluster. A clustering analysis was performed by weighted gene correlation network analysis, which resulted in 40 clusters of genes co-expressed together. (**b**) The bar graph shows the proportion of ATAF1 and ANAC055 targets in each cluster, including genes targeted by both; clusters with significant enrichment of ATAF1 and ANAC055 target genes are shaded (*P* < < 0.001); only two clusters (10 and 13) are highly enriched with ATAF1 targets; clusters 10 and 21 are significantly enriched for ANAC055, and clusters 10, 13 and 21 are enriched for genes that are targeted by both transcription factors.

Genes with similar expression patterns often share similar functions and are potentially regulated by the same transcription factors. In order to identify genes co-regulated with *ATAF1* in response to heat priming, a co-expression analysis was performed using the transcriptomic data of *ATAF1*-OE, *ataf1–4* mutant and WT plants, under control condition and upon exposure to heat. A weighted correlation network analysis was used to find modules of highly correlated genes. This method is commonly used to investigate relationships between genes and to identify gene candidates for further analysis^46^. The co-expression analysis revealed 40 modules in our data set (Fig. 6a). These modules contained groups of genes expressed in a similar way. It is likely that genes regulated directly by ATAF1, in response to heat, display similar expression patterns; hence, we took publicly available data on genes that have already been identified to be potentially targeted by ATAF1 (in general, not just by heat stress) and queried if these genes were present in our co-expression modules. The publicly available data of the potential direct targets were generated using a DAP-seq assay^33^. Putative target genes were designated as having a DAP-seq peak within the first 1000 bp upstream of the transcription start site. Thus, we compared the list of genes that are proposed direct targets of ATAF1 (from O’Malley et al.^33^) with the genes that are clustered in the co-expression analysis; we noticed that the potential targets of ATAF1 were overrepresented in co-expression clusters 10 and 13 (Fig. 6b). These clusters needed further investigation because they likely contained direct target genes of ATAF1, in response to heat stress. The clusters were further interrogated with the proposed TF targets identified using the DAP-seq assay developed by O’Malley et al.^33^. We found that three of the clusters (10, 13, 21) showed significant enrichment for genes targeted by both ATAF1 and ANAC055, suggesting that the genes in these clusters might be co-regulated by the two NACs (Fig. 6b).

### ATAF1 and ANAC055 appear to have a common role in thermomemory

**Figure 7:**
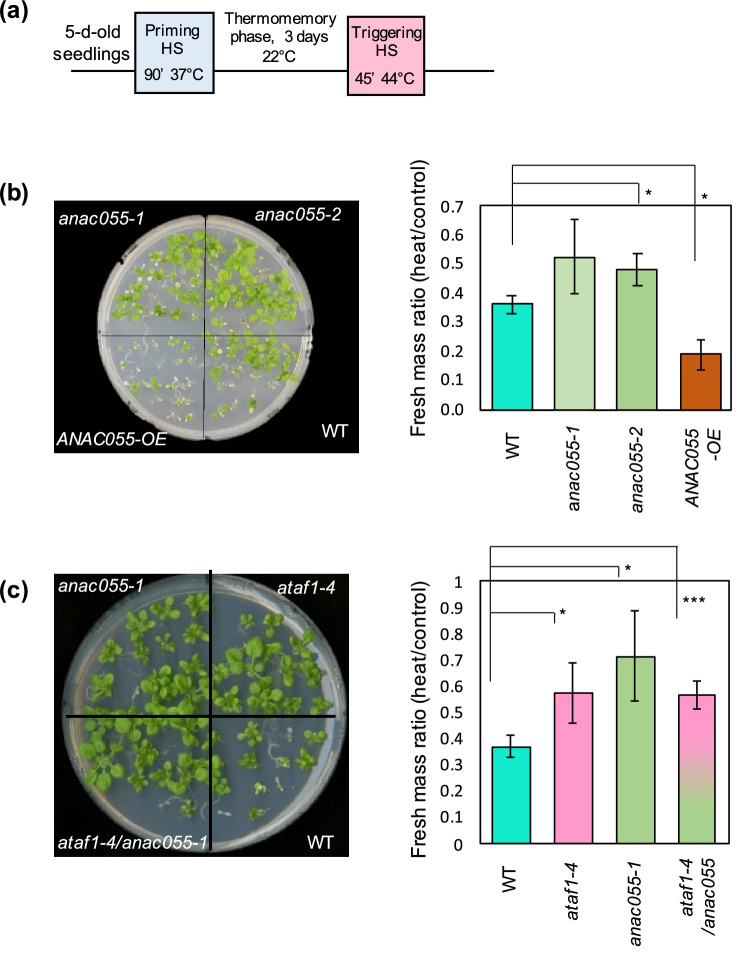
Thermomemory is improved in the *ataf1–4*/*anac055* double-knockout mutant compared to WT. (**a**) Schematic representation of HS regime. (**b**) Thermomemory phenotype of *ANAC055-*OE, *anac055-1* and *anac055-2* mutants, and WT plants. (**c**) Thermomemory phenotype of *ATAF1*-OE, *ataf1–4* mutant, *ataf1–4*/*anac0*55 double-mutant, and WT plants. Seedlings were exposed to the HS regime shown in (**a**). Left panels: seedlings photographed 14 days after exposure to the triggering heat stress. The phenotype of one representative replicate of at least three (**b**) or five (**c**) independent biological replicates is shown. Right panels: seedling fresh mass upon heat stress, compared to control plants (no heat stress). Error bars represent standard deviation, which was calculated from three biological replicates, where each replicate was the average mass of 18–19 seedlings. Significant differences between transgenic lines and WT plants were calculated using Student’s *t*-test; * if *P* < 0.05; *** if *P* < 0.001.

Our co-expression analysis showed that ATAF1 and ANAC055 had a large number of shared co-expressed targets in response to HS (Supplementary Table S5). We therefore tested if ANAC055 is also functionally involved in regulating thermomemory. To this end, we assessed the thermomemory phenotype of transgenic lines with altered expression of *ANAC055* (Fig. 7a,b). Two *anac055* mutants (*anac055-1* and *anac055-2*) were tested, and both showed a substantial increase in fresh mass ratio, survival, and chlorophyll content compared with WT, while plants overexpressing *ANAC055* showed a decrease in those parameters (Fig. 7b, Supplementary Fig. S4). These results indicate a negative role for ANAC055 in the regulation of thermomemory, similar to that of ATAF1. To test whether or not ATAF1 and ANAC055 worked in a fully or partially redundant manner, we generated an *ataf1*/*anac055* double mutant and exposed seedlings to HS and compared their phenotypes with the phenotypes of the corresponding single-gene mutants. The fresh mass ratio of the *ataf1*/*anac055* double-mutant was higher than that of the WT, but not significantly different from that of the single-gene knockout mutants *ataf1–4* and *anac055-1* (Fig. 7c). This result indicates that ATAF1 and ANAC055 require each others function to control thermomemory.

## Discussion

HS is one of the major abiotic stresses negatively affecting plant growth and reproduction globally. The impact of HS on crop production increases with climate change. Prior exposure to mild HS (heat priming) enables plants to acquire and maintain tolerance to HS by establishing a cellular memory of a prior HS (thermomemory)^1–4^. Here, we studied the role of NAC TFs in thermomemory. We found that several NACs substantially responded at their expression level after heat stress and that the changes in expression persisted during the memory phase. These findings are similar to those reported for the expression pattern of other thermomemory-associated genes during the memory phase (such as *HSFA2*, *HSA32*, and *JUB1*)^8,9,24^.

We then analyzed the expression of NAC TFs after the triggering stimulus (second heat stress) to investigate if their expression was affected by a priming treatment. Our data show that NAC expression was altered after the triggering stimulus, indicating the presence of transcriptional memory that controls their expression. A similar expression pattern has previously been reported for drought memory, where the expression of a subset of DEGs is altered after a second drought stress. Ding et al.^47^ classified the memory genes into four distinct groups, each showing different expression patterns: genes with increased (or decreased) expression levels after a first stress, and a further increased (or decreased) level after a second stress, which they, respectively, denoted as (+/+, or −/−); ‘revised memory genes’ that are induced after a first stress and then decreased after the second stress (+/−), or the opposite (−/+). Many NACs showed an expression pattern similar to those reported by Ding et al.^47^.

We found that *ATAF1* (*ANAC002*) expression increases rapidly right after the priming HS, and then decreases to below control levels during the memory phase (Fig. 1; cluster 2). We also found that *ataf1* knockout mutants were more tolerant to heat stress than the wild type, revealing a negative regulatory role of *ATAF1* in HS memory. ATAF1 was previously identified as an important regulator of senescence and the response to drought stress^29,31,48^. It has been well established that ATAF1 promotes senescence by directly regulating the expression of two TFs involved in senescence and chloroplast maintenance: *ANAC092* (*ORE1*) and *GOLDEN2-LIKE1* (*GLK1*)^31^. ATAF1 also regulates drought response by regulating the expression of *9-CIS-EPOXYCAROTENOID DIOXYGENASE 3* (*NCED3*), which encodes a key enzyme of ABA biosynthesis^31,49^.

We used RNA-seq analysis to identifying early transcriptional changes controlled by ATAF1. Several mechanisms (genes) have been previously identified to be involved in basal heat tolerance: they include ROS detoxification, lipid signaling, and activation of heat shock proteins and heat shock factors^8–10,27,50^. Our data found similar responses in *ATAF1*-OE, *ataf1–4* mutant and WT plants, where these mechanisms were similarly activated.

Co-expression analysis has been established as a powerful tool to identify TF target genes^51,52^. For instance, Jensen et al.^49^ performed a co-expression analysis across publicly available microarray datasets and identified 25 genes co-expressed with *ATAF1*. The promoter regions of these *ATAF1* co-expressors were significantly enriched for ATAF1 binding sites. We performed a co-expression analysis and combined this information with publicly available data from a DAP-seq approach^33^. We found that four co-expression clusters were enriched with predicted ATAF1 target genes; and those were also enriched for target genes of another NAC TF, ANAC055. We found many target genes commonly regulated by ATAF1 and ANAC055, suggesting at least partial overlap between the two TFs with respect to their regulatory capacity. Often, TFs act in a redundant manner. For example, the transcription factors WRKY11 and WRKY17 operate as negative regulators of basic resistance to *Pseudomonas syringae* pv*.* tomato (*Pst*)^50^. The loss of function of WRKY11 increases the resistance toward *Pst*. An expression analysis of selected genes in single and double mutants revealed that both TFs modulate transcriptional changes in response to pathogen infection; however, each one of them acted either specifically or in a partly redundant manner, depending on the specific target gene^50^. In our work, we identified ATAF1 and ANAC055 as negative regulators of thermotolerance. Phenotypic and gene expression data of *ataf1* and *anac055* single and double mutants showed a partial regulatory redundancy between *ATAF1* and *ANAC055*.

Our results suggest that ATAF1 and ANAC055 work both as negative regulators of thermomemory because (i) the single *ataf1* and *anac055* mutants as well as the double-mutant plants have a similar phenotype in thermomemory, (ii) they appear to be co-expressed, and (iii) their potential target genes overlap. Further protein-interaction studies are needed to test if ATAF1 and ANAC055 also interact physically, for instance as heterodimers or if they are only connected via their gene-regulatory networks. This interaction needs to be investigated in a temporal and spatial context to evaluate its relevance for thermomemory.

It has recently been suggested that the heat-inducible transcription factor HSFA2 is involved in thermomemory, particularly regulating HS memory genes in the shoot apical meristem (SAM), suggesting that the establishment of thermomemory involves organ-specific mechanisms^53,54^. Our results show that in *ataf1–4* lines, a higher proportion of plants displayed full recovery, i.e. seedlings and cotyledons remained green compared to WT plants. In plants that only partially recovered, cotyledons remained bleached and the developing true leaves emerged green (Supplementary Fig. S4). The green emerging leaves suggest that mechanisms that protect the SAM, where new leaves develop, play a role in thermomemory.

Most of the current understanding of thermotolerance and thermopriming is based on Arabidopsis seedlings. However, there have been some limited studies performed to assess the thermopriming in crop plants. For instance, heat priming of winter wheat during the stem-elongation, bolting and anthesis stages significantly enhanced grain-yield under heat stress during the grain filling phase^55^. Another mechanism for improving thermotolerance may be through root endophytes which appear to enhance thermotolerance of crops by impacting the expression of thermomemory associated gene *HSFA2*^56^. Our study shows the importance of two genes, *ATAF1* and *ANAC055*, as negative regulators of thermomemory in seedlings; it remains to be tested if later growth or seed yield are also modified under recursive HS. At least for seedlings tested here, a biomass penalty under non-stress conditions was not observed. Once a better understanding of thermomemory is established in model organisms and crops, genome editing technology could be employed to enhance crop performance in the field in anticipated future climatic conditions.

## Materials and methods

### Plant materials

*Arabidopsis thaliana* ecotype Col-0 was used as wild type and obtained from the European Arabidopsis Stock Center (NASC) (http://arabidopsis.info/). The *ATAF1-OE*, *ataf1-2* (SALK-057618) and *ataf1–4* (GABI-Kat GK565H08) seeds were described previously in Garapati et al.^31^. Seeds of the T-DNA insertion lines of *ANAC055* (*anac055-1*; SALK_014331 and *anac055-2*; SALK_011069) were obtained from The European Arabidopsis Stock Centre (NASC) seed collection (http://arabidopsis.info/). The *ANAC047* transgenic lines, i.e., *ANAC047-*OE (*35S::ANAC047-GFP*) and the two knockout lines (T-DNA insertion lines *shyg-1*/SALK-066615 and *shyg-2*/GABI-Kat GK-343D11) were previously described by Rauf et al.^57^. Seeds of *ANAC013*-OE and knockdown (*anac013-kd*) lines were provided by Prof. Frank van Breusegem (Ghent University, Ghent, Belgium) and described in De Clercq et al.^58^.

### Plasmid construction and plant transformation

For *35S::ANAC055* (*ANAC055-*OE), the *ANAC055* open reading frame was amplified by PCR from Arabidopsis Col-0 leaf cDNA and inserted into pUni/V5-His-TOPO (Invitrogen). The cDNA was then cloned via added *Pme*I-*Pac*I sites into a modified pGreen0229-35S plant transformation vector^59^. The resulting pGreen0229-35S::*ANAC055* vector was electroporated into *Agrobacterium tumefaciens* strain GV3101 and transformed into Arabidopsis wild type via floral dip. To construct *pATAF1::ATAF1-GFP*, a genomic fragment encompassing the 1.5-kb *ATAF1* promoter and the *ATAF1* coding region (without the translation stop codon) was inserted upstream of the green fluorescence protein coding sequence (in-frame fusion) in plasmid pK7FWG2,0^60^. The cloning replaced the CaMV *35S* promoter present in the plasmid with the *ATAF1* promoter. The *ataf1* knockout complementation line was generated by transforming the *ataf1–4* mutant^31^ with the *pATAF1::ATAF1-GFP* construct.

### Double mutant

The *ataf1*/*anac055* double-knockout mutant was generated by crossing the homozygous *ataf1–4* mutant with the homozygous *anac055-1* mutant. Plants from the F2 generation were PCR-screened to select homozygous double-mutant plants. The list of primers used for genotyping is given in Supplementary Table S6.

### Plant growth conditions

Arabidopsis seeds were surface-sterilized by washing them with 70% ethanol for 2 min; the ethanol solution was then replaced with 20% sodium hypochlorite and seeds were incubated in the solution for 20 min, with continuous shaking. Subsequently, seeds were washed four times with sterile water and left to dry on sterilized filter paper, in a laminar clean bench. Approximately equal numbers of seeds (around 500 seeds) were germinated on small petri dishes containing half-strength Murashige and Skoog (MS) medium supplemented with 1% (w/v) sucrose. For stratification, plates were kept in a dark cold room (8 °C) for 2 days before placing them in a growth chamber (photoperiod 16 h/8 h: light/dark at 22.5 °C, under 120 μmol m^−2^ s^−1^ photon irradiance). Five-day-old seedlings were used for HS assays.

### Heat stress treatment

For NAC expression profiling, 5-day-old Arabidopsis seedlings were exposed to a HS regime. Plants were primed with mild heat stress at 37 °C for 90 min in a growth chamber, and subsequently returned back to normal growth condition to recover for 2 days to assess their thermomemory. Plants were then challenged with a triggering HS of 44 °C for 45 min. Arabidopsis seedlings were collected during the memory phase, and after the triggering stimulus, at the time points indicated in Fig. 1. Whole seedlings collected from one single plate were considered as one biological replicate. Control samples (without treatment) were collected at the same time to reduce circadian effects. Three biological replicates per treatment, per time point, and per genotype were analysed. Samples were directly frozen in liquid nitrogen, and stored at − 80 °C until further analysis.

### Chlorophyll content

The Chlorophyll content was measure using the protocol of Hiscox and Israelstam^61^. Frozen seedling of ten were immersed in 0.5 mL preheated dimethylsulfoxid (DMSO, 65 °C) and incubated at 65 °C for 20 min. For the measurement the extract was diluted 1:10 or 1:100 with DMSO. Absorbance was recorded at 663 nm and 645 nm, respectively, for chlorophyll *a* and *b*. The following formula^62^ was used to calculate the concentration of chlorophyll *a*, *b* and total chlorophyll as mg g^−1^ fresh mass (FM):

$${Chl}_{a }\left(\text{g L} ^{-1}\right)=0.0127\left({OD}_{663}\right)-0.00269\left({OD}_{645}\right),$$

$${Chl}_{b}\left(\text{g L}^{-1}\right)=0.229\left({OD}_{645}\right)-0.00488\left({OD}_{663}\right),$$

$${Total}_{Chl}\left(\text{g L}^{-1}\right)= {0.202Chl}_{a}+ {0.00802Chl}_{b},$$

$${Total}_{Chl} \left(\text{mg g}^{-1}\right)=\frac{20.2{Chl}_{a}+8.02{Chl}_{b}}{FM}.$$

Data was normalized to the respective control of each genotype.

### RNA isolation, cDNA synthesis and qRT-PCR for NAC expression profiling

For expression profiling of NAC genes, total RNA was extracted using Trizol (Ambion, Life Technologies), followed by purification of RNA using columns of the PureLink RNA Mini kit (Ambion, Life Technologies) according to the manufacturer’s instructions. RNA concentration was measured using a NanoDrop spectrophotometer (Thermo Scientific); genomic DNA was digested using the TURBO DNA-free Kit (Ambion) according to the manufacturer’s instructions. The absence of genomic DNA contamination was confirmed by qRT-PCR, using intron-specific primers of control gene *AT5G65080* (for primer sequences, see Supplementary Table S6). cDNA was synthesized from 4 µg high-quality RNA, using the Revert Aid H Minus First Strand cDNA Synthesis Kit (Thermo Scientific), following the instructions of the manufacturer.

The qRT-PCR reaction was performed using SYBR Green master mix (Applied Biosystems): cDNA 0.5 μL, SYBR Green master mix (2×) 2.5 μL, forward and reverse primer mix (each 0.5 μM) 2 μL. The reactions were incubated in an ABI PRISM 7900 HT sequence-detection system (Applied Biosystems) using the following protocol: temperature was first set at 95 °C and kept constant for 10 min, and then underwent a series of 40 cycles of 95 °C for 15 s and 60 °C for 1 min. *ACTIN2* (*AT3G18780*) was used as the reference gene for data analysis. The primer platform of the 104 Arabidopsis NAC TF genes was established using the QuantPrime tool^63^. Primer sequences are given in Supplementary Table S6. After 40 reaction cycles, a melting curve was generated by increasing the temperature from 60 to 95 °C, with a ramp speed of 1.9 °C min^−1^, to verify the amplification of the desired products. The efficiency of the reaction was estimated using LinRegPCR software, as described by Caldana et al.^64^.

Data were analyzed using the comparative Ct method, as described in Kamranfar et al.^65^. Briefly, the delta Ct value (ΔCt) was calculated by normalizing each Ct value with the Ct value of the reference gene *ACTIN2*. Then, the level of gene expression was expressed as the difference between an arbitrary value of 40 and the ΔCt value (a high 40-ΔCt value indicates a high gene expression level). A threshold of 1.5-fold change was used to select differentially expressed genes.

### RNA isolation and RNA-seq

For the RNA-seq analysis, 5-day-old Arabidopsis seedlings of the three genotypes were used: WT, *ataf1–4* mutant, and *ATAF1*-OE. After treatment with the heat priming temperature (37 °C for 90 min), seedlings were harvested at time points 0 h, 1 h and 4 h during the memory phase.

The transcriptomes of the three genotypes WT, *ataf1–4* and *ATAF1-*OE was analyzed using RNA-seq technology. Total RNA was isolated from 5-day-old Arabidopsis seedlings of the three genotypes. Whole seedlings collected from each plate (pool of at least 10 seedlings) were considered as one biological replicate. Three biological replicates per treatment, per time point, and per genotype, were used, which made 72 samples in total. RNA was isolated using Direct-zol RNA MiniPrep Plus (Zymo Research, CA, USA) according to the manufacturer’s instructions. Quality was assessed using a Bioanalyzer (Agilent Technologies, Waldbrann, Germany), and quantity was determined using Qubit (Invitrogen, Life Technologies). High-quality RNA (3 µg) was used to prepare libraries for sequencing. RNA-sequencing 150-bp paired-end (PE) libraries were prepared from mRNA-enriched samples, using the NEB Next Ultra DNA Library Prep Kit for Illumina sequencing (New England Biolabs), according to the manufacturers’ instructions. A total of 12 samples were pooled in one lane of the flow cell. RNA was sequenced, using an Illumina HiSeq4000 machine, at KAUST Core Labs.

### RNA-seq data analysis

HISAT was used to align the RNA-seq reads onto the genome of *Arabidopsis thaliana* (TAIR10)^66^. Only sequencing reads that mapped uniquely on the genome were retained. Htseq-count was used to count the number of sequencing reads of the transcripts^67^. VOOM, a transformation method that allows the use of RNA-seq data for the linear model framework, was used to calculate the transcript abundance, and quantile normalization was used to normalize the abundance distribution between samples^68^. EdgeR and LIMMA^69,70^ were used as a framework for calculating the differentially expressed genes. R, a general statistical environment, was used for data management, analyses, and generating figures^71^.

The customized linear contrast was set up to calculate the differential expression between genotypes, or temperatures. More specifically, second-order interaction effects were calculated using the contrast framework^70^. For example, a calculation could be made to quantitatively assess the changes in gene expression under heat, in comparison with the control for *ataf1* and WT: (*heat*_*ataf1*_* − control*_*ataf1*_*) − *(*heat*_*WT*_* − control*_*WT*_*)*. All statistical significance cut-offs were adjusted for multiple testing by controlling the False Discovery Rate (Benjamini-Hochberg) at 0.05^72^ using “decideTests” (…, method = “global”, adjust.method = “BH”, p.value = “0.05”, lfc = 0.

For all genes and samples, a weighted gene correlation network analysis was performed to identify groups of co-expressed genes^46^, using default parameters. The soft thresholding power parameter was set at 6, which is the value, based on graphical examination, that the optimal network conditions are met (maximum scale-free fit index and minimum mean connectivity). For multi-dimensional scaling, the *cmscale* function in R was used.

Transcription factor binding sites (TFBS) acquired via DAP-seq as reported by O’Malley et al.^33^ were extracted from the publicly available plant cistrome database. DAP-seq binding motif peaks were extracted in narrowPeak format and mapped to the Arabidopsis genome. Distance from peak location to closest transcription start site (TSS) was then computed via the bedtools ‘closest’ command^73^, where TSS locations were taken from the TAIR 10 annotation file^74^. Putative ATAF1 and ANAC055 target genes were defined as having at least one DAP-seq peak within the first 1000 bp upstream of the respective TSSs. Statistical analysis for enrichment of target genes within the co-expression clusters was performed in R, using a hypergeometric test for overrepresentation^71^.

### Legislation statement

The experimental research and field studies on plant materials comply with relevant institutional, national, and international guidelines and legislation.

## Supplementary Information

Supplementary Figure S1.

Supplementary Figure S2.

Supplementary Figure S3.

Supplementary Figure S4.

Supplementary Table S1.

Supplementary Table S2.

Supplementary Table S3.

Supplementary Table S4.

Supplementary Table S5.

Supplementary Table S6.

## Data Availability

RNA sequencing data are available from the NCBI Bioproject database under ID SUB8978006 (https://ngdc.cncb.ac.cn/search/?dbId=gsa&q=SUB8978006). All other data generated or analysed during this study are included in this published article and its Supplementary Information files.
