# Supplementary material for: NAC transcription factors ATAF1 and ANAC055 affect the heat stress response in Arabidopsis
Source: Sci Rep. 2022 Jul 4;12:11264. doi: 10.1038/s41598-022-14429-x (PMC9253118; doi:10.1038/s41598-022-14429-x)
Supplement: Supplementary file 1 — Supplementary Figure S1. [file 41598_2022_14429_MOESM1_ESM.pdf]

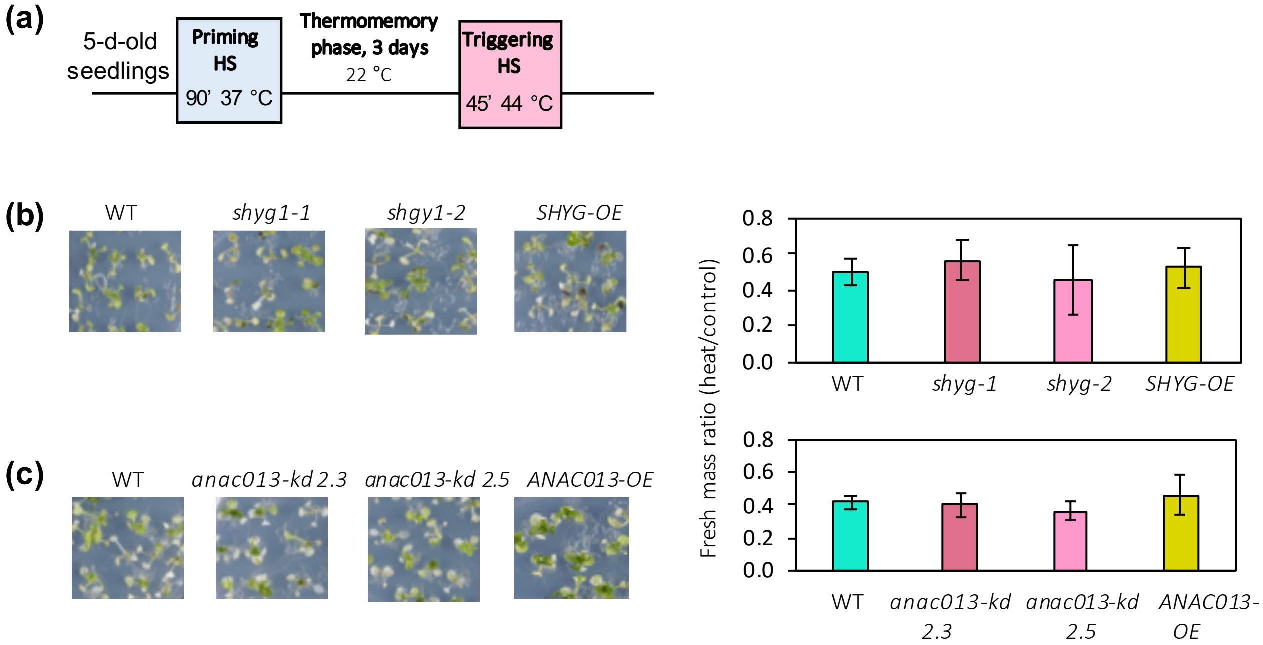

Supplementary Figure S1. Functional analysis of selected NACs in thermomemory.
(**a**) Schematic representation of the heat stress (HS) regime applied to assess HS thermomemory. Thermomemory phenotype of plants overexpressing genes, or their respective mutants, and quantification of fresh mass for (**b**) *SHYG1* (*ANAC047*), (**c**) *ANAC013*. Photos were taken 14 days after heat stress. Seedling fresh mass in HS is compared to control plants (no heat stress). One representative replicate of at least three independent biological replicates is shown. Error bars represent the standard deviation, which was calculated from three biological replicates, where each replicate represents the average mass of 18 seedlings. No significant differences were detected.
