# Supplementary material for: NAC transcription factors ATAF1 and ANAC055 affect the heat stress response in Arabidopsis
Source: Sci Rep. 2022 Jul 4;12:11264. doi: 10.1038/s41598-022-14429-x (PMC9253118; doi:10.1038/s41598-022-14429-x)
Supplement: Supplementary file 2 — Supplementary Figure S2. [file 41598_2022_14429_MOESM2_ESM.pdf]

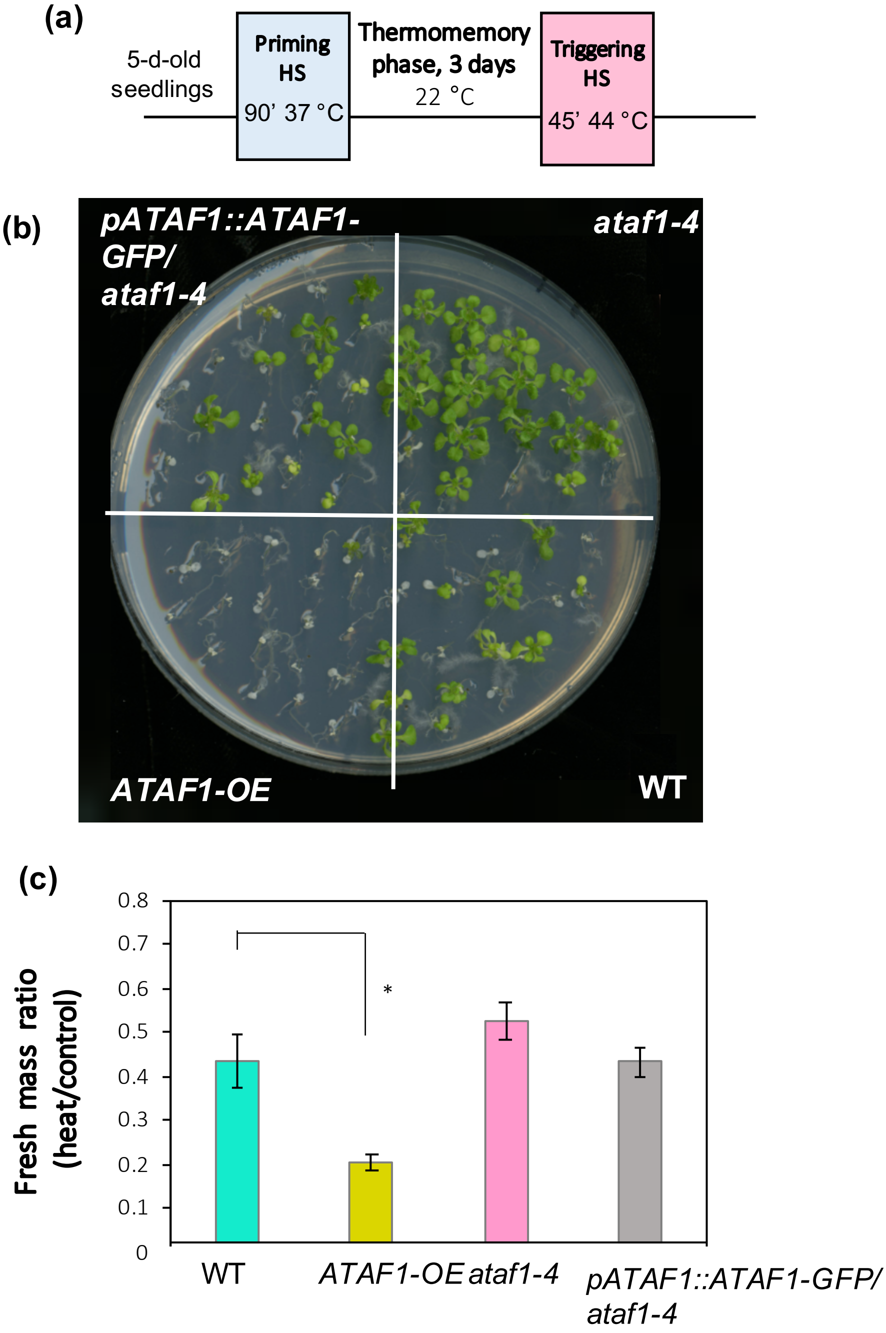

Supplementary Figure S2. Thermomemory phenotype of the *ATAF1* complementation line.

The *ataf1-4 mutant* was transformed with the *ATAF1p::ATAF1* construct. (**a**) Schematic representation of the heat stress (HS) regime applied to asses HS thermomemory. (**b**) Thermomemory phenotype of *pATAF1::ATAF1-GFP/ataf1-4* transgenic plants. Seedlings of *ATAF1*-OE, *ataf1-4*, *ATAF1p::ATAF1/ataf1-4*, and WT were exposed to HS regimes schematically shown in panel (a); photos were taken 14 days after the second HS. The phenotype of one representative replicate of at least three independent biological replicates is shown. (**c**) Seedling fresh mass after HS compared to control plants (no heat stress). Error bars represent the standard deviation, which was calculated from three biological replicates, where each replicate is the average mass of 19 seedlings. Significant differences among transgenic lines and WT plants were calculated using Student's *t*-test; * indicates P-value < 0.05.
