# Supplementary material for: NAC transcription factors ATAF1 and ANAC055 affect the heat stress response in Arabidopsis
Source: Sci Rep. 2022 Jul 4;12:11264. doi: 10.1038/s41598-022-14429-x (PMC9253118; doi:10.1038/s41598-022-14429-x)
Supplement: Supplementary file 3 — Supplementary Figure S3. [file 41598_2022_14429_MOESM3_ESM.pdf]

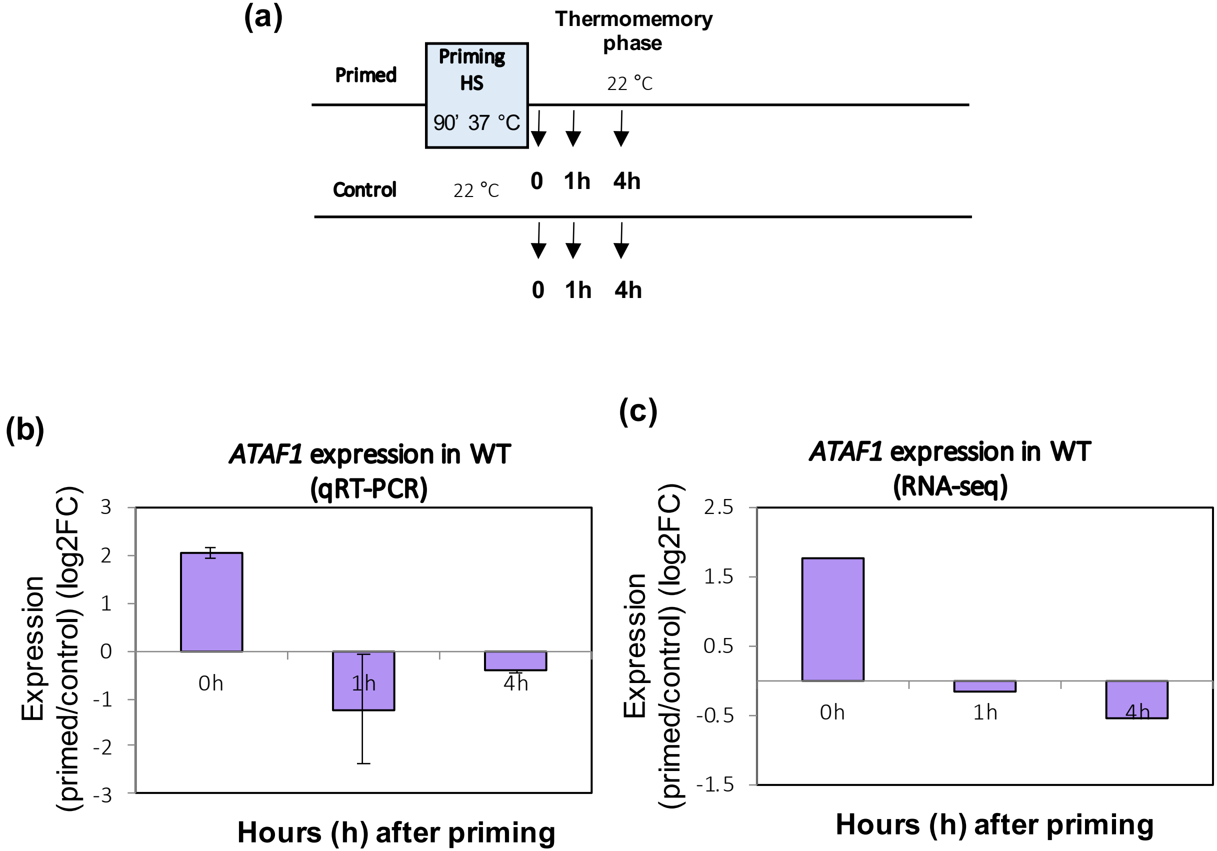

Supplementary Figure S3. Confirmation of *ATAF1* expression in WT plants after priming treatment using qRT-PCR and RNA-seq.
(**a**) Schematic representation of the heat stress (HS) regime applied to asses *ATAF1* expression in response to priming. (**b**) Expression data from qRT-PCR. Expression is shown as $\log_2$-fold change between HS and control. (**c**) Expression data from RNA-seq analyses.
