# Supplementary material for: NAC transcription factors ATAF1 and ANAC055 affect the heat stress response in Arabidopsis
Source: Sci Rep. 2022 Jul 4;12:11264. doi: 10.1038/s41598-022-14429-x (PMC9253118; doi:10.1038/s41598-022-14429-x)
Supplement: Supplementary file 4 — Supplementary Figure S4. [file 41598_2022_14429_MOESM4_ESM.pdf]

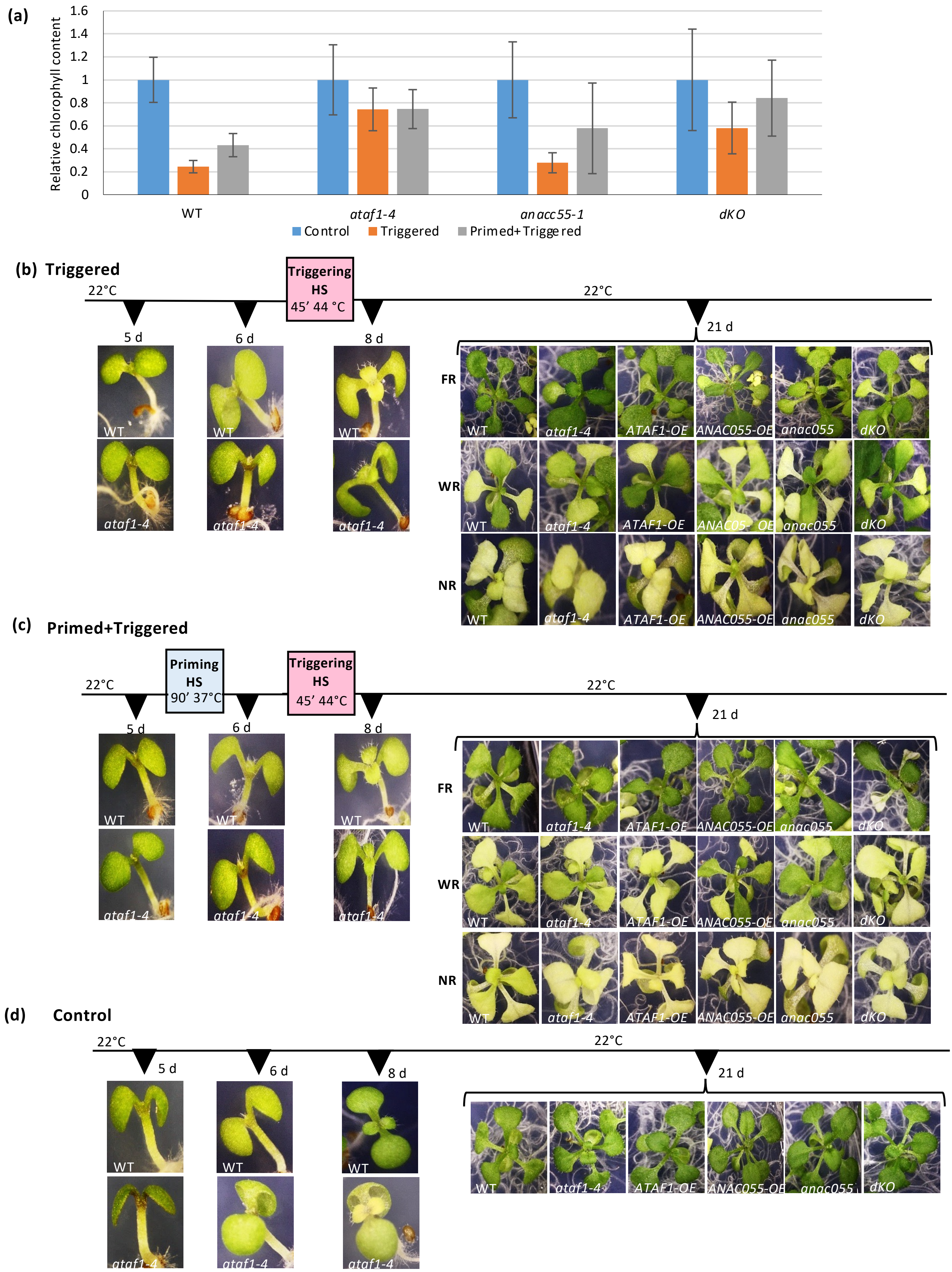

Supplementary Figure S4. Phenotypes in response to heat treatments.

(a) Chlorophyll content of WT, *ataf1-4*, *anac055-1 and* dKO (*ataf1-4*/*anac055*) plants. Seedlings were exposed to the HS regime primed+triggered, just triggered, or control, without a HS treatment. Seedlings were analysed 14 d after exposure to the triggering stimulus. Chlorophyll content of the transgenic lines was normalized to the respective control's chlorophyll content; n was at least 3 independent biological replicates, each biological replicate containing 10 seedlings. Standard error of the mean is displayed. **(b)**, **(c)** and **(d)** Phenotype of plants with overexpression or knock out of *ATAF1* and *ANAC055*. Seedlings of WT, *ataf1-4*, *anac055-1*, *ATAF1-OE*, *ANAC055-OE* and dKO (*ataf1-4*/*anac055-1*) were exposed to the corresponding heat stress (HS) regimes. The phenotype of one representative replicate of at least three independent biological replicates is shown for full recovery (FR), weak recovery (WR) and no recovery (NR).
